# Repeated Moderate Noise Exposure in the Rat—an Early Adulthood Noise Exposure Model

**DOI:** 10.1007/s10162-015-0537-5

**Published:** 2015-07-11

**Authors:** Paula Mannström, Mette Kirkegaard, Mats Ulfendahl

**Affiliations:** Department of Neuroscience, Karolinska Institutet, Retzius väg 8, B1:5, SE-171 77 Stockholm, Sweden

**Keywords:** repeated noise exposure, hearing, threshold shift, amplitude, rat

## Abstract

In this study, we investigated the effects of varying intensity levels of repeated moderate noise exposures on hearing. The aim was to define an appropriate intensity level that could be repeated several times without giving rise to a permanent hearing loss, and thus establish a model for early adulthood moderate noise exposure in rats. Female Sprague-Dawley rats were exposed to broadband noise for 90 min, with a 50 % duty cycle at levels of 101, 104, 107, or 110 dB sound pressure level (SPL), and compared to a control group of non-exposed animals. Exposure was repeated every 6 weeks for a maximum of six repetitions or until a permanent hearing loss was observed. Hearing was assessed by the auditory brainstem response (ABR). Rats exposed to the higher intensities of 107 and 110 dB SPL showed permanent threshold shifts following the first exposure, while rats exposed to 101 and 104 dB SPL could be exposed at least six times without a sustained change in hearing thresholds. ABR amplitudes decreased over time for all groups, including the non-exposed control group, while the latencies were unaffected. A possible change in noise susceptibility following the repeated moderate noise exposures was tested by subjecting the animals to high-intensity noise exposure of 110 dB for 4 h. Rats previously exposed repeatedly to 104 dB SPL were slightly more resistant to high-intensity noise exposure than non-exposed rats or rats exposed to 101 dB SPL. Repeated moderate exposure to 104 dB SPL broadband noise is a viable model for early adulthood noise exposure in rats and may be useful for the study of noise exposure on age-related hearing loss.

## INTRODUCTION

Hearing impairment is one of the most common disabilities in modern society, according to National Institute on Deafness and Other Communication Disorders (NIDCD, 2010). Of the US adult population, 17 % report some degree of hearing loss, a number which increases with age. Genetic factors contribute significantly to the functional impairment, but external environmental factors such as exposure to intense noise (Oishi and Schacht 2011), ototoxic drugs (Rybak et al. 2007), or solvent chemicals (Johnson and Nylen 1995) are equally important and may exacerbate the hearing loss. Increased awareness of risk factors has resulted in protective measures, and today, the working population is to a much lesser extent subjected to intense noise at the workplace. However, we are living in an increasingly noisy world where individuals are exposed to moderate everyday noise from their leisure activities, e.g., loud music from earphones and high volume at the cinemas, gyms, rock concerts, etc. While these repeated noise exposures, sometimes of quite high intensities, usually do not elicit a detectable loss of hearing, their long-term effects on auditory function are not well explored. It has, however, been suggested that these exposures will adversely affect the progression of age-related hearing loss (Stockwel et al. 1969; Johnsson and Hawkins 1972; Mostafapour et al. 1998; Dalton et al. 2001).

The effect of noise exposure on hearing has been studied extensively in mammals (Liberman and Dodds 1984; Slepecky 1986; Wang et al. 2002). Typically, intense noise results in a permanent hearing loss, while noise at lower intensities causes a temporary hearing loss. In both cases, the hearing loss reflects oxidative stress and structural changes at the level of the hearing organ. In conditions of permanent threshold shift, mechanical damage to the inner and outer hair cells as well as the afferent nerve fibers has been reported (for a review, see Ohlemiller 2008). The cellular damage also leads to a secondary degeneration of neurons (Kujawa and Liberman 2006), and moreover, *stria vascularis* and fibrocytes in the spiral ligament are affected (Hirose and Liberman 2003). In cases of a temporary threshold shift, structural observations include degeneration of afferent nerve terminals, detachment of the hair cell stereocilia from the overlying tectorial membrane, and buckling of pillar cells (Nordmann et al. 2000). Those changes are explained as direct consequences of moderate noise exposures. Other consequences are swelling of cochlear nerve terminals, which is an acute response to noise caused by glutamate excitotoxicity. Loss of presynaptic ribbons and postsynaptic terminals of the inner hair cells precedes the swelling (Kujawa and Liberman 2009; Lin, et al. 2011; Ruttiger et al. 2013). These changes remain even when the hearing thresholds return to normal levels. Interestingly, in these studies the auditory brainstem response (ABR) amplitudes did not recover, which were believed to be a consequence from the histopathological changes of the peripheral nerve. Also, the biochemical composition becomes altered after noise exposures. For example, both excitatory and inhibitory neurotransmission-related proteins and neuroplasticity markers were altered in the rat auditory pathway after intense noise exposure (Browne et al. 2012).

The degree of hearing loss after noise exposure depends on the total energy content of the noise, which in turn depends on the noise intensity, exposure time, possible repetition rate, and interval length as described in a review article by Clark (Clark 1991). By integrating periodic rest periods, some protective effects were observed in terms of less hearing loss and histological damage. In a study by Clark and colleagues (Clark et al. 1987), chinchillas were exposed to octave band noise, 95 dB sound pressure level (SPL), 6 h per day for 36 days. The initial threshold shift of 35–45 dB recovered after a few days of exposure to a shift of 10–15 dB. In another study (Hamernik et al. 1994), more intense noise exposures (107, 113, 119, and 125 dB SPL) were used at two different exposure protocols in chinchillas. One group was exposed 6 h per day for 20 days while another group was exposed during a non-interrupted schedule of 24 h per day for a total of 5 days. Both groups thus received the same total exposure energy. Hearing recovered better and histological data revealed a milder hair cell loss for the group exposed on an interrupted schedule over a longer period. This data suggests a toughening of the hearing organ when rest periods are introduced to the noise exposure protocol.

Kujawa and Liberman showed that noise exposure in young CBA/CaJ mice exacerbated the age-related hearing loss in aging animals, compared to non-exposed age-matched controls, even if the initial increased threshold shift after the noise exposure was reversible (Kujawa and Liberman 2006). Interestingly, when a group of older animals was exposed to the same type of noise, the initial effects on hearing thresholds as well as the structural degeneration of the neurons and the sensory cells were not as severe, as in the younger animals. This study indicates that a reversible hearing loss at a young age may accelerate the age-related hearing loss and that younger animals are more sensitive to noise than older ones. It is therefore of interest and clinical importance to study also the effect of moderate noise exposure in younger animals and the long-term effects on hearing.

The aim of the present study was to develop an animal model mimicking the exposure of young adults to non-damaging noise levels, by exposing rats in early adulthood to repeated moderate noise at levels that do not cause a permanent hearing loss. The effects of different intensity levels were evaluated by monitoring changes in ABR. Female Sprague-Dawley rats were exposed to noise at selected intensities at 6-week intervals, up to six times or until they displayed a permanent threshold shift. The groups that did not show permanent threshold shifts even after six exposures were subjected to a high-intensity noise exposure to assess the influence of repeated noise preexposures on the subsequent susceptibility to noise-induced pathology. The main objectives of the present study was to explore how an increase in intensity levels of repeated noise exposures changed the threshold shift in an animal from temporary to permanent. This included monitoring the functional changes of ABR threshold shifts, reduced amplitudes, and delayed latencies. Finally, it was of interest to investigate whether repeated exposures to moderate noise altered the susceptibility to more intense noise.

## MATERIAL AND METHODS

### Subjects

A total of 40 female albino Sprague-Dawley rats (Harlan laboratories, the Netherlands) starting from the age of 9 weeks were used in this study. Animals were kept three to four in wire-meshed cages with free access to food and water in the same holding unit on a 12/12-h day and night cycle. All animal procedures followed the local ethical regulations at Karolinska Institutet and were in consistence with national regulations for care and use of animals, approval numbers N33/07, N12/10, and N300/11.

### Experimental Design

**FIG. 1:**
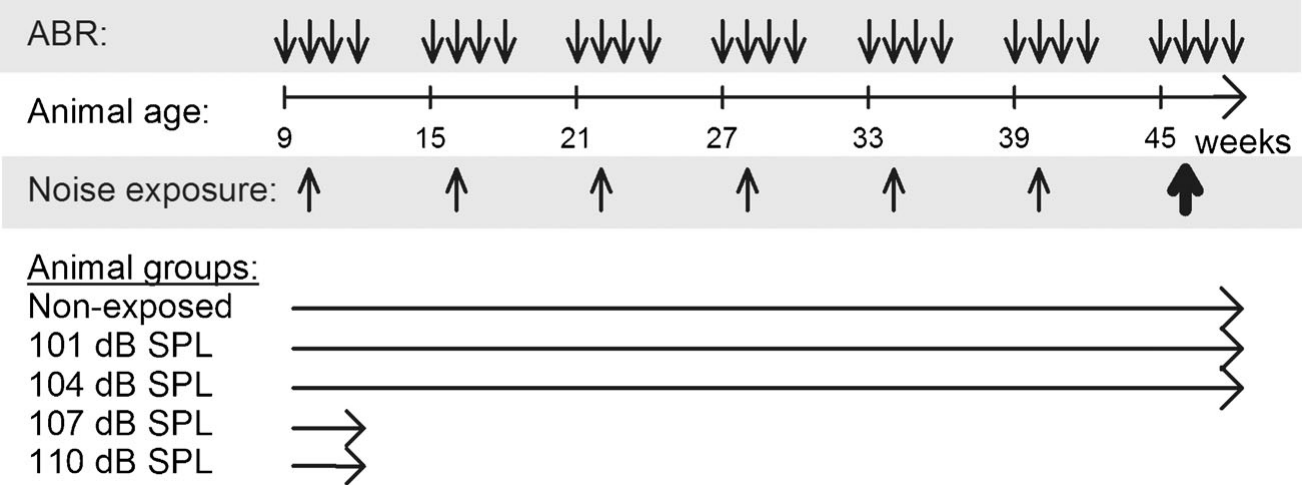
The experimental design showing time points for ABR recordings (*downward arrows*) 1 week before the first noise exposure and 24 h, 1 week, 2 weeks, and 5 weeks after each of the repeated moderate noise exposures (*thin upward arrows*) followed by high-intensity noise exposure (*thick upward arrow*). The *horizontal arrows* after the five animal groups correspond to time spent in the experiment.

The animals were divided into groups exposed to brief periods of noise at different intensities: 101 (*n* = 8), 104 (*n* = 8), 107 (*n* = 7), and 110 (*n* = 7) dB SPL. Age-matched animals that were not exposed served as a control group (*n* = 10). Exposures were repeated every 6 weeks for up to six times or until the animals received a permanent hearing loss (Fig. 1). Baseline hearing thresholds were assessed by recording ABR 1 week before the first noise exposure. The ABRs were then recorded 24 h, 1 week, 2 weeks, and 5 weeks after each repeated exposure. For the non-exposed control animals, ABRs were recorded once every 6 weeks from 9 weeks of age. The groups which displayed no permanent threshold shift even after six repetitions and the non-exposed group were finally exposed to a high-intensity noise, known to cause permanent threshold shifts in this animal strain (unpublished data), and the hearing was assessed 24 h and 1 and 2 weeks following the intense exposure.

### Noise Exposures

Two freely moving rats were simultaneously exposed in individual cages, centrally placed side by side inside a soundproof box with the dimensions 104 × 78 × 112 cm (width × depth × height). The noise, generated using Brüel & Kjær 3560-C PULSE hardware and a LAB 300 amplifier with PULSE LabShop Version 13.1.0.246 software (Brüel & Kjær, Denmark), was presented through a transducer (Model 2482, JBL, LA, USA) with a Beyma TD-360 horn (Acustica Beyma, Spain) centrally positioned inside the box, 85 cm above the animal cages. The noise was calibrated using a microphone (Brüel & Kjær, Denmark) placed between the cages and adjusted to the desired level before each exposure. Prior to the experiments, the noise level was recorded at each of the four corners of the cages and shown to vary by ±2 dB SPL.

For repeated moderate noise exposures, the rats were exposed to broadband free-field noise, 2–20 kHz, at a 50 % duty cycle (500 ms of exposure each second) for 90 min at intensity levels of 101, 104, 107, or 110 dB SPL. Groups that showed no permanent threshold shift after six repetitions (i.e., the 101 and the 104 dB SPL groups) and the non-exposed control group were then exposed to a high-intensity noise of 110 dB SPL (narrowband free-field noise, 3.2 kHz bandwidth) for 4 h.

### Hearing Assessment by Recording Auditory Brainstem Responses

The animals were anesthetized using an intraperitoneal injection of a 2.7 ml/kg mixture of 1 ml Hypnorm®, 1 ml Dormicum (5 mg/ml Midazolam®), and 2 ml sterile water. ABR recordings were randomly made in either left or right ear, and the same ear was subsequently used throughout the study. Before the first ABR recording was performed, the tragus cartilage was removed in order to place the microphone close to the eardrum. The eardrums were inspected using an otoscope, and animals with middle ear infections were excluded. After placing the animal on a non-electric heating pad inside a soundproof box, subcutaneous needle electrodes were positioned at the vertex (active), behind the recorded ear (reference), and in the hind leg (ground). Acoustic stimuli were generated by a Tucker-Davis Technologies system (BioSig 32 Ver 3.12, Tucker-Davis Technologies, FL, USA), presented through an electrostatic speaker (EC1, Tucker-Davies Technologies, FL, USA), which was connected to the ear canal of the rat. Frequency-specific tone bursts of 3.5, 7, 14, and 28 kHz were presented at a duration of 2, 1, 0.5, and 0.25 ms for the respective frequencies and with a 1, 0.5, 0.25, and 0.125 ms raise and fall time.

**FIG. 2:**
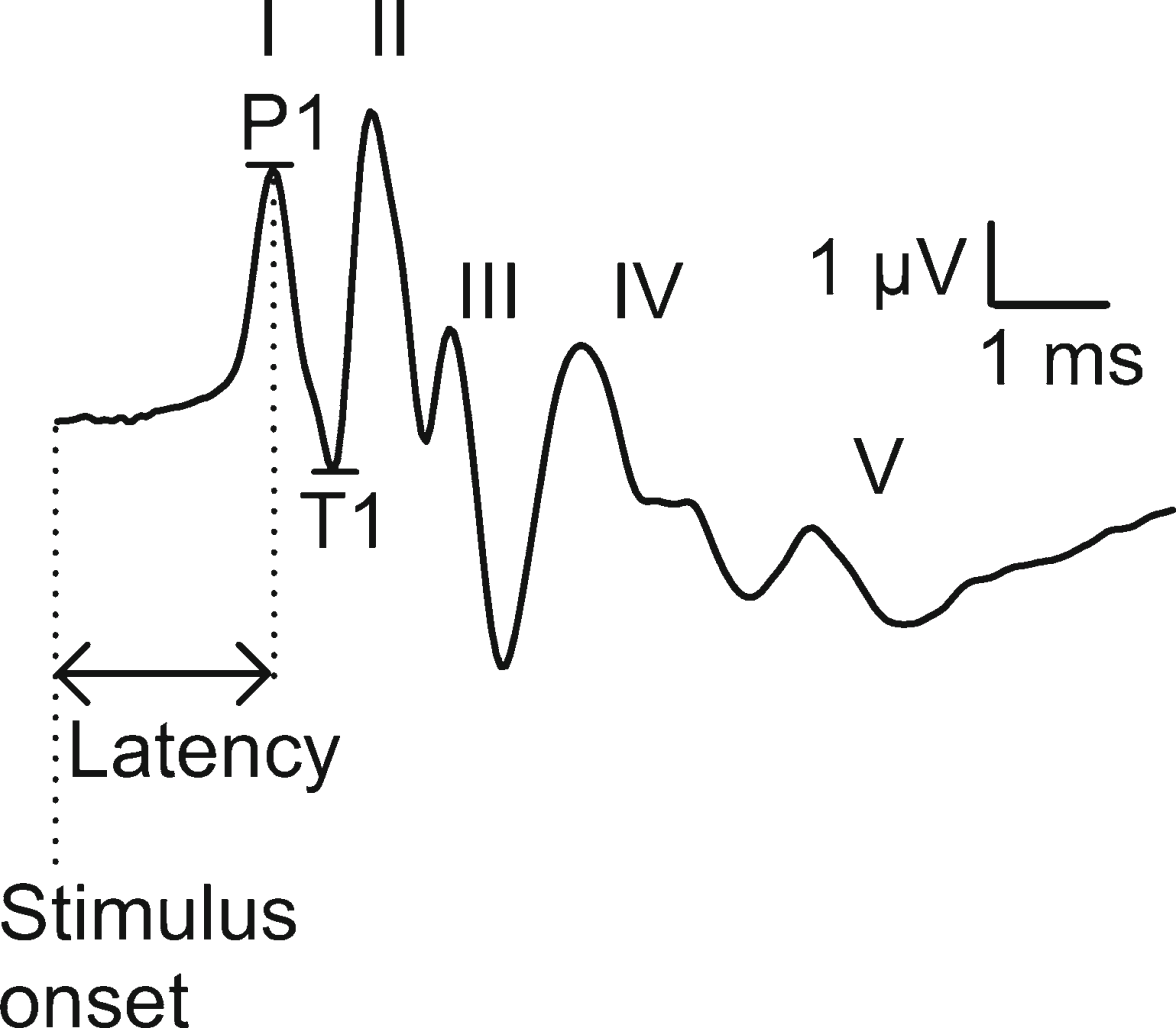
ABR waveforms marked *I*–*V* from a young rat with normal threshold. ABR wave I _*p-p*_ amplitudes (μV) were calculated as the peak to peak difference from wave I peak (P1) to its trough (T1). The *dotted lines* indicate the latency, i.e., the time from the onset of the stimuli to the peak of wave I.

ABR thresholds were determined as the lowest stimulus level that produced a reproducible response for ABR wave II, which was visualized at the same latency after an average of 2000 recordings. The second wave was chosen as it is the largest wave of the rat’s ABR and thus most readily monitored, also when the stimulus level is lowered. The stimulus intensity was lowered in steps of minimum 5 dB until the threshold was reached. The threshold shifts were calculated by subtracting the recorded thresholds with the baseline threshold recorded before the first exposure. Amplitudes and latencies from ABR wave I were extracted at an acoustic stimulus of 80 dB SPL after an average of 1000 recordings. Wave I is generated from the auditory nerve and is a measure of the number and the synchrony of the neurons firing. The amplitudes were measured as the peak to peak _*(p-p)*_ amplitude difference from the peak of wave I (P1) to its trough (T1) (Fig. 2) (Alvarado et al. 2012). The latencies were defined as the time between the onset of the stimulus and the peak of wave I (P1) (Fig. 2). The relative amplitudes and latencies were calculated by dividing the value at each time point with the value from the first measurement. Measurements were made at all measured frequencies.

### Statistics

Differences between groups were compared statistically by one-way ANOVA followed by a Holm-Sidak comparison, and comparisons within the groups over time were analyzed using one-way repeated measures ANOVA followed by Holm-Sidak comparison on repeated measurements. When normality test failed, Kruskal-Wallis one-way ANOVA on ranks using Dunn’s method as an all pair-wise multiple comparison procedure was used. The significance level was set to *P* < 0.05. Data analysis was performed using SigmaPlot for Windows Version 11.0. All data are presented as mean ± standard deviation (SD).

## RESULTS

### A Single Exposure at 107 or 110 dB SPL Elicited a Permanent Threshold Shift

**FIG. 3:**
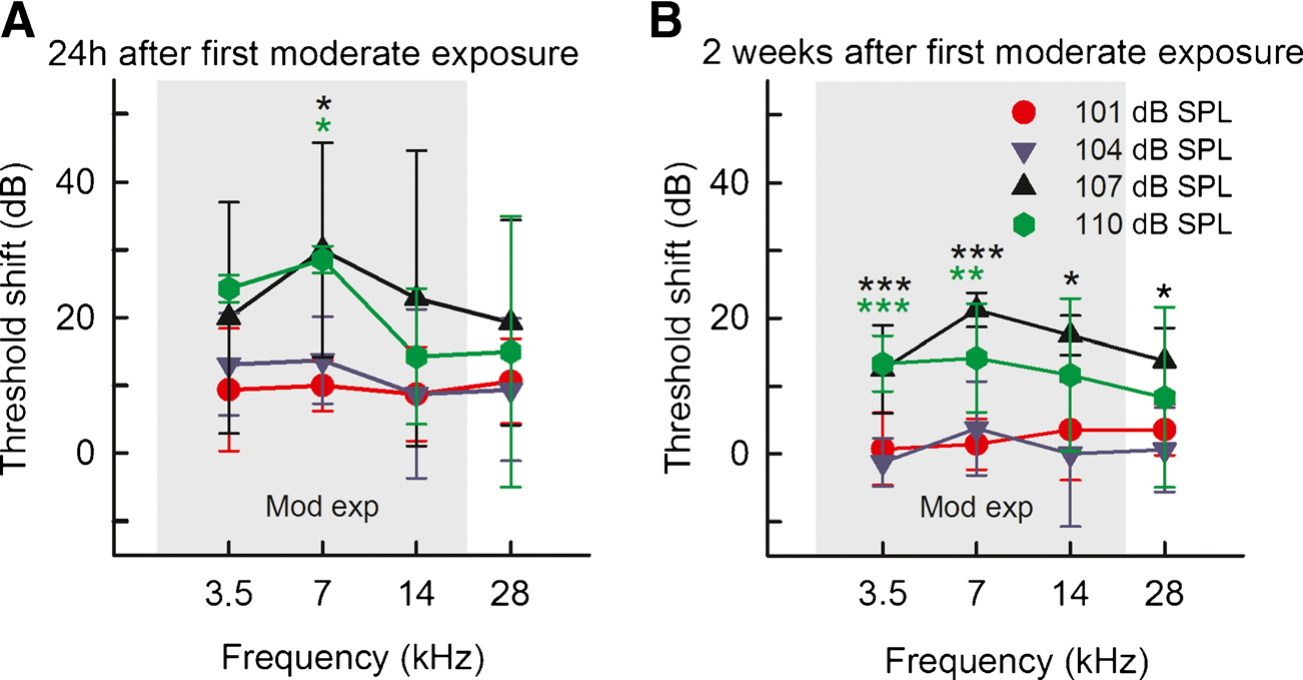
**A** ABR threshold shifts 24 h after the first moderate noise exposure. Significantly increased thresholds of 15–28 dB for the groups exposed to 107 and 110 dB SPL compared to the groups exposed to 101 and 104 dB SPL. **B** Two weeks later, the threshold shifts for the 107 and 110 dB SPL groups were reduced to 11–20 dB but were still significantly higher than for the groups exposed to 101 and 104 dB SPL, where the thresholds had returned to baseline levels. The area marked *Mod exp* is the frequency span for the moderate exposure. *P < 0.05, **P < 0.01, ***P < 0.001, *colors* correspond to exposure groups, which significantly differ from the other groups.

After the first exposure to moderate noise, the rats’ auditory function was affected. For animals exposed to 107 or 110 dB SPL, ABR threshold shifts were around 20 dB (for all tested frequencies) at 24 h after the first exposure (Fig. 3A). At 7 kHz, these groups differed significantly from animals exposed to 101 and 104 dB SPL (one-way ANOVA on ranks, *H*_(3)_ = 18.6, *P* < 0.05 for both 110 dB compared to 101 and 104 dB SPL as well as 107 dB SPL compared to 101 dB SPL). After 2 weeks, all threshold shifts decreased in all subjects, but the animals exposed to 107 and 110 dB SPL continued to display an increased threshold shift of about 15 dB at all tested frequencies (Fig. 3B), and the shifts were thus considered permanent. The threshold shifts were significantly higher (one-way ANOVA at 3.5 kHz; *F*_(3)_ = 16.2, *P* < 0.001) both for 107 and 110 dB SPL compared to 101 and 104 dB SPL. At 7 kHz; *F*_(3)_ = 12.6, *P* < 0.001 for 107 dB SPL compared to 101 and 104 dB SPL, *P* = 0.001 for 110 dB SPL compared to 101 dB SPL and *P* = 0.004 for 110 dB SPL compared to 104 dB SPL. At 14 kHz one-way ANOVA on ranks, *H*_(3)_ = 9.77, *P* < 0.05 for 107 dB SPL compared to 104 dB SPL. At 28 kHz one-way ANOVA on ranks, *H*_(3)_ = 8.71, *P* < 0.05 for 107 dB SPL compared to 104 dB SPL) than those obtained in animals exposed to the lower intensities (101 and 104 dB SPL), which had a mean threshold shift of about 2 dB. The animals exposed to 104 or 101 dB SPL differed neither from each other nor from the non-exposed control group regarding threshold shifts.

### 104 dB SPL Was the Highest Intensity Level that Could Be Repeated Six Times Without Causing a Permanent Threshold Shift

**FIG. 4:**
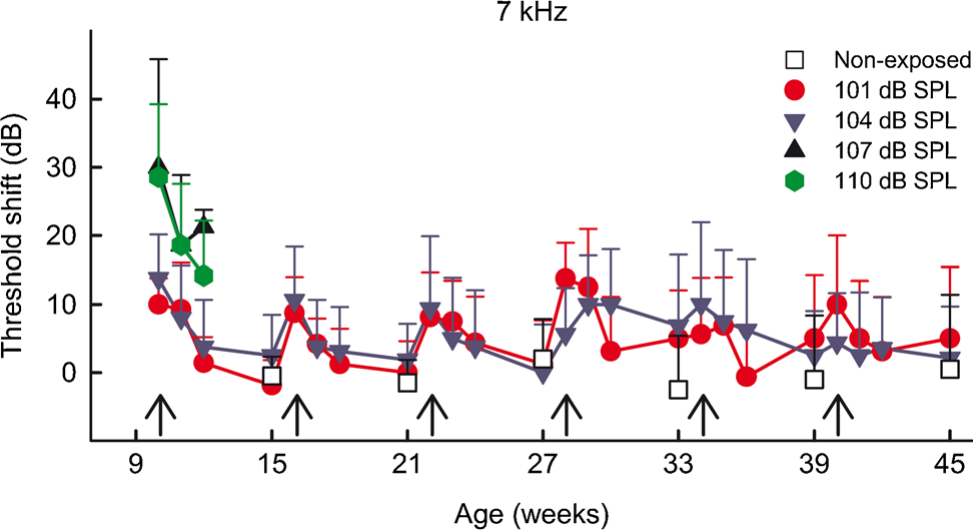
Mean ABR threshold shifts at 7 kHz for the animal groups exposed to different intensity levels (101, 104, 107, and 110 dB SPL) at 24 h, 1 week, 2 weeks, and 5 weeks after each repeated noise exposure. In the age-matched, non-exposed control group, ABRs were recorded every 6 weeks. *Arrows* indicate the time points for the repeated exposures.

The animals exposed to intensity levels of 107 and 110 dB SPL displayed permanent threshold shifts after the first exposure, and these groups were thus not further exposed. The remaining groups, exposed to intensity levels of 101 and 104 dB SPL, could be exposed for at least six repetitions without achieving permanent threshold shifts (average threshold shifts from a representative ABR frequency from 7 kHz is shown in Fig. 4). Each exposure produced a temporary threshold shift of approximately 10 dB at 24 h and 1 week after the noise exposure, but after 2 weeks, the ABR thresholds returned to baseline levels. All tested frequencies displayed the same pattern. The non-exposed control group displayed stable thresholds throughout the whole period, thus confirming that no age-related hearing loss affected the hearing during the experiment. The animals were 45 weeks old at the time point for the last ABR recording.

### ABR Amplitudes Were Reduced in Animals with Recovered Thresholds

**FIG. 5:**
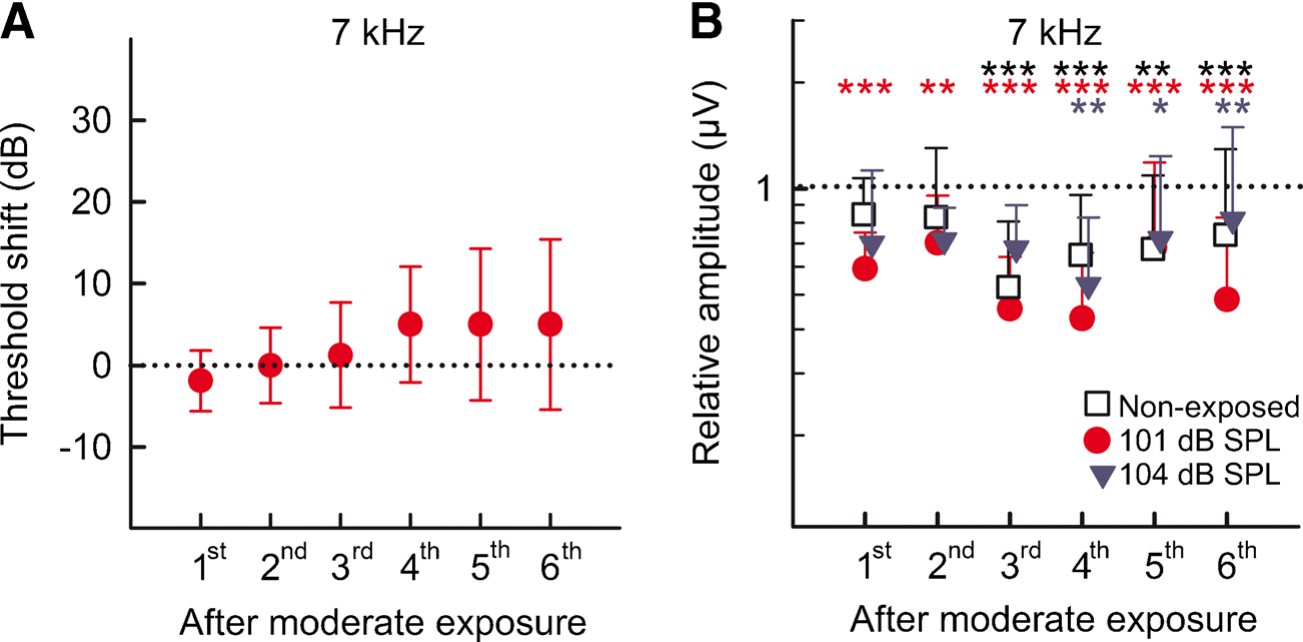
**A** No significant differences in ABR threshold shifts were seen over time when comparing ABRs 5 weeks after each of the repeated moderate noise exposures. ABRs recorded at 7 kHz for the group exposed to 101 dB SPL is shown. **B** Relative ABR wave I _*p-p*_ amplitudes were significantly reduced over time compared to the amplitudes from the first ABRs before the first exposure. *Dotted lines* indicate baseline values. **P < 0.01, ***P < 0.001, compared to baseline values within the same group, *colors* correspond to exposure groups.

For the groups exposed to the lower intensity levels (101 and 104 dB SPL), the effect of the repeated moderate noise exposures were explored in detail 5 weeks after each exposure (i.e., every 6 weeks) and compared to the non-exposed control group (Fig. 5). Threshold shifts as well as the ABR wave I _*p-p*_ amplitudes and latencies were compared between the groups to investigate the impact of the different intensity levels. Effects of the repeated exposures and age changes were also studied by comparing ABRs within the same group over time. The ABR threshold shifts in all three groups varied only with ±5–10 dB 5 weeks after each exposure, both between the groups and over time within the groups, which was within the limit of individual differences (the 101 dB SPL group at representative measurements from 7 kHz is shown in Fig. 5A). Neither the repeated moderate exposures nor age had any effect on the threshold shifts. When comparing ABR wave I _*p-p*_ amplitudes, no significant differences were detected between the groups at any time point. However, significant reductions were detected within all three groups over time. The 101 dB SPL group displayed significantly reduced amplitudes after the first exposure and at all time points thereafter (one-way repeated measures ANOVA, *F*_(6)_ = 7.34, Holm-Sidak test for comparison over time, representative measurements from 7 kHz shown in Fig. 5B and Table 1). The ABR amplitudes of the group exposed to 104 dB SPL were also reduced, but significant changes were not detected until the measurement after the fourth exposure (one-way repeated measures ANOVA, *F*_(6)_ = 2.78, Holm-Sidak test for comparison over time, representative measurements from 7 kHz shown in Fig. 5B and Table 1). Interestingly, in the non-exposed control group significantly reduced amplitudes were detected at the time point when the exposure groups were measured after their third exposure (one-way repeated measures ANOVA, *F*_(6)_ = 4.909, Holm-Sidak test for comparison over time, representative measurements from 7 kHz shown in Fig. 5B and Table 1). Amplitudes were thus reduced with age rather than as an effect of the repeated moderate noise exposures.

**TABLE 1:** Relative ABR wave I _*p-p*_ amplitudes after each repeated moderate noise exposure (values divided with the value from the first measurement)

After moderate exposure	Non-exposed	101 dB SPL	104 dB SPL
	*n* = 10, *F*_(6)_ = 4.91	*n* = 8, *F*_(6)_ = 7.34	*n* = 8, *F*_(6)_ = 2.78
1	0.83 ± 0.22, n.s*.*	0.59 ± 0.16, *P* < 0.001	0.70 ± 0.43, n.s.
2	0.82 ± 0.47, n.s.	0.70 ± 0.26, *P* = 0.01	0.72 ± 0.17, n.s.
3	0.52 ± 0.28, *P* < 0.001	0.46 ± 0.18, *P* < 0.001	0.67 ± 0.22, n.s.
4	0.64 ± 0.31, *P* = 0.001	0.43 ± 0.23, *P* < 0.001	0.53 ± 0.30, *P* = 0.002
5	0.67 ± 0.41, *P* = 0.007	0.68 ± 0.51, *P* < 0.001	0.72 ± 0.52, *P* = 0.012
6	0.73 ± 0.51, *P* = 0.001	0.48 ± 0.34, *P* < 0.001	0.82 ± 0.69, *P* = 0.005

Average levels ± standard deviations with significance level (one-way repeated measures ANOVA, Holm-Sidak test compared to the baseline value from the same exposure group) for the different groups, ABRs at 7 kHz with a stimulus level of 80 dB SPL

*n.s.* not significant

The ABR latencies were more stable over time, and only in few of the measurements, after several exposures, prolonged latencies were detected in the group exposed to 104 dB (data not shown). Also here, no statistical differences were found between the groups.

### Repeated Exposures at 104 dB SPL Resulted in Animals Appearing More Resistant to High-Intensity Noise Exposure

**FIG. 6:**
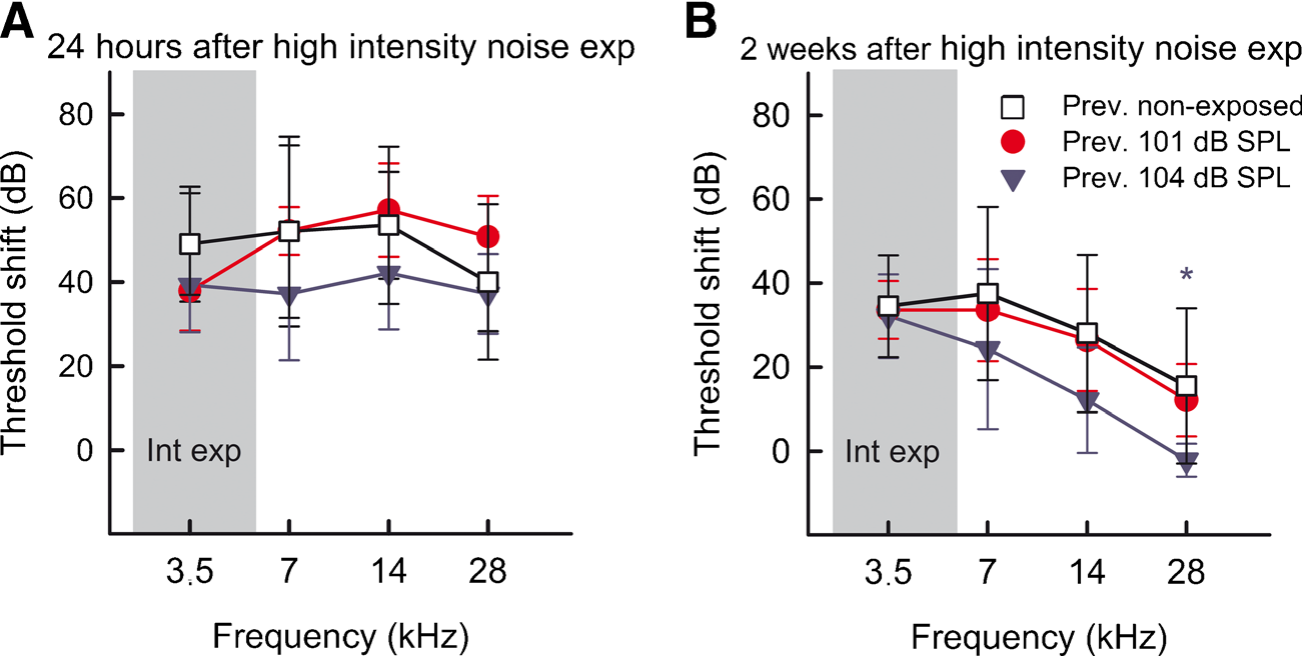
ABR threshold shifts after high-intensity noise exposure using a 4-h-long narrowband noise exposure of 110 dB SPL. **A** At 24 h after the acoustic overstimulation, the groups with previously non-exposed animals and animals repeatedly exposed at 101 and 104 dB SPL displayed threshold shifts of about 40–50 dB. The group previously exposed at 104 dB SPL had slightly lower threshold shifts, but no significant differences were detected. **B** Two weeks after the acoustic overstimulation, the threshold shifts had become permanent at about 20–40 dB. The group previously exposed at 104 dB SPL displayed lower threshold shifts than the other groups, which were significant at 28 kHz when compared to the previous non-exposed group. The area marked *Int exp* indicates the frequency span of the high-intensity noise exposure. *P = 0.013 between the groups of previously non-exposed and exposed to 104 dB SPL.

Even though no permanent hearing thresholds were observed in the groups repeatedly exposed to 101 and 104 dB SPL, a possible change in noise susceptibility was investigated. The animals, including the previously non-exposed group, were thus exposed to 110 dB SPL narrowband noise for 4 h, an exposure known to induce permanent threshold shifts in this rat strain. The resulting threshold shifts peaked after the more intense exposure to around 40–50 dB at 24 h following the exposure (Fig. 6A), decreased with time, and was permanent at about 20–40 dB after 2 weeks (Fig. 6B). The highest threshold shift was found at the lower frequencies, close to frequency of the noise exposure. The group previously exposed repeatedly to 104 dB SPL displayed slightly lower threshold shifts compared to the other groups at all recorded time points after the acoustic overstimulation, statistically significant after 2 weeks at 28 kHz when the thresholds where back to normal levels (one-way ANOVA, *F*_(2)_ = 3.95, compared to the previously non-exposed group; Holm-Sidak test, *P* = 0.013). Neither the wave I _*p-p*_ amplitudes nor latencies of the ABR wave differed at any time point between any of the groups (data not shown).

## DISCUSSION

In this study, we investigated the functional effects of moderate noise exposures at different intensity levels in early adulthood of the female Sprague-Dawley rat in order to determine the levels that could be repeated several times without causing permanent threshold shifts. In the present model, 104 dB SPL was the highest exposure intensity that could be repeated up to six times without causing a permanent change in hearing thresholds. In addition, exposures at this level provided a certain protective effect from subsequent acoustic overstimulation of higher-intensity noise exposure. We suggest that repeated exposures of 104 dB SPL can be used as a model for studying the influence of early adulthood noise exposures on age-related hearing loss.

A broadband noise exposure with intensity levels of both 101 and 104 dB SPL, for 90 min at a 50 % duty cycle, could be repeated up to six times with no permanent effect on hearing thresholds. The same type of exposures with intensity levels of 107 or 110 dB SPL produced a permanent threshold shift following the first exposure. The exposures started when the female rats were young adults (10 weeks old) and continued until the rats were middle-aged (about 10 months of age). At this age, the non-exposed rats displayed normal hearing thresholds without any signs of an incipient age-related hearing loss. It is possible that the first exposure could fall within an early vulnerable period for the rat. However, previous studies indicate that the most sensitive period for the rat are around 20–25 days (Lenoir et al. 1979, Saunders 1982), which corresponds to the period of functional and structural maturation of the cochlea. In the present study, the first exposure started at 10 weeks of age and, at that time, the cochlea was fully mature and the early vulnerable period had passed. The onset of age-related hearing loss in this strain (Sprague-Dawley) occurs around 12 months of age. The Sprague-Dawley rat is an outbred strain and was chosen for this study because of its similarities to humans regarding diversity in sensory and motor function during aging (Altun et al. 2007) and with the aim to be used as a future model strain. The age-related histological changes of the inner ear in the Sprague-Dawley rat have previously been studied in our laboratory. Loss of sensory hair cells and a diminished volume of stria vascularis were the major age-related changes with increasing age (Mannstrom et al. 2013). In the future, both histological as well as biochemical changes need to be further explored after repeated moderate noise exposures and increasing age.

Threshold shifts, wave I _*p-p*_ amplitudes, and latencies were determined from ABRs that were conducted 5 weeks after each of the repeated moderate noise exposures in the animal groups that did not demonstrate a permanent threshold shift after the exposures, e.g., the groups exposed to 101 and 104 dB SPL, together with the non-exposed group. The thresholds were normal both over time and between all groups. The ABR wave I _*p-p*_ amplitudes decreased significantly over time for all groups, including the control group, but did not significantly differ between the groups. This indicates that the spiral ganglion activity, reflected in the magnitude of the ABR amplitude, was reduced due to age rather than exposure. The decline was significant compared to baseline levels after the first exposure for the group exposed to 101 dB SPL and after the fourth exposure for the group exposed to 104 dB SPL. The animals in the control group were almost 7 months (27 weeks) when their amplitudes were significantly reduced; if compared to humans, it would correspond to early mid-life. In human studies, the peak to peak amplitude of the ABR waves also becomes reduced at early ages, when hearing thresholds are still normal (Konrad-Martin et al. 2012). Consistent with other animal studies (gerbils and rhesus monkeys), we found that amplitudes were reduced with age irrespectively of threshold shifts (Boettcher et al. 1993; Torre and Fowler 2000). In addition to increasing age, the most common reason for a decline in amplitude is noise exposures. In a study where guinea pigs were exposed to octave band noise (4–8 kHz, for 2 h at 106 dB SPL) eliciting a temporary threshold shift, permanent reduced amplitudes as well as structural changes were observed (Lin et al. 2011). In another study, using repeated noise exposures, the same pattern of altered amplitudes preceding permanent threshold shift changes was presented (Wang and Ren 2012). In that study, CBA/CaJ mice were exposed three times to a 100 dB SPL noise (8–16 kHz, 2 h, 2 weeks apart). The ABR wave I _*p-p*_ amplitudes were reduced after the first noise exposure, but only after the third exposure the threshold shift became permanent. In contrary to these studies, we did not detect any significantly altered amplitudes caused by the exposures creating temporary threshold shift. The reduced amplitudes were instead considered as a cause of increasing age.

The latencies of the ABR wave I peak appeared almost completely normal throughout the whole study, consistent with the hearing thresholds. Other studies have reported that prolonged latencies were only found in aging subjects if the hearing threshold was increased (Boettcher 2002), suggesting that the intensity levels of our noise exposures were not high enough to affect the ABR latencies. However, both noise and aging can cause changes in ABR amplitudes and eventually threshold shifts. It would be of interest to further explore possible structural differences at the level of the afferent nerve terminals after repeated moderate exposures.

Since the repeated noise exposures did not give rise to increased ABR thresholds, we were interested in exploring possible changes in the animals’ hearing using a more intense noise exposure. The thresholds were, as expected, increased 24 h after the exposure, and after 2 weeks, the threshold shifts were considered permanent for all groups (including the previously non-exposed control group). The highest threshold shift was found at frequencies close to the frequency span of the exposure-noise. The group previously exposed repeatedly to 104 dB SPL had slightly lower hearing thresholds than the other groups, which were significant at 28 kHz compared to the non-exposed group, and moreover, at this frequency the thresholds had returned to normal. This group of animals had previously also displayed a decrease of ABR amplitudes later than the other groups. These results indicate that this intensity level of repeated moderate noise exposures made the animals somewhat more resistant to intense noise exposure later in life. Two types of “training” phenomena are described in the literature, both of them reducing inner ear susceptibility to noise. In one type, a low level of continuous non-damaging noise is used prior to a more intense noise exposure and is referred to in the literature as “sound conditioning” (Canlon et al. 1988; Yoshida and Liberman 2000). The second type, named “toughening,” is observed following an interrupted time schedule, with exposure of animals to a noise that creates a temporary threshold shift (Miller and Watson 1959; Pukkila et al. 1997). Several exposures made the ears “toughened” resulting in smaller threshold shifts as the number of exposures increased. Neither sound conditioning nor toughening fully matches our experimental protocol with moderate 6-week interval of repeated noise exposures. Either intensity levels used in these studies were much lower (between 55 and 95 dB SPL) than in our exposures or the duration of the exposure were longer (typically for more than a week) with different repetition rates from days to several months. However, the mechanisms underlying sound conditioning or toughening may very well be the same as those involved in our model with repeated moderate noise exposures. One explanation for the beneficial effects could be triggering of several protective systems, for example the endogenous antioxidant system (Jacono et al. 1998) or production of neurotrophic factors (Nam et al. 2000), which are believed to protect cochlear structures from future damage.

The long-term auditory effects of non-damaging sound exposures throughout the life span are relatively unknown. The present animal model using repeated moderate noise exposures to mimic human early adulthood exposures may be used to explore these effects and their influence on age-related hearing loss. In daily life, individuals are regularly exposed to moderate sound levels during both working hours and leisure activities. The exposures may lead to a slight, often not detected, temporary hearing loss, which declines after a few days. Our observations indicate that these short-term effects do not impair hearing sensitivity. However, it should be noted that little is known of the long-term effects on onset and severity of age-related hearing loss. Other investigators have studied the effects of noise exposure at young age on age-related hearing loss (Kujawa and Liberman 2006, 2009), but they have primarily investigated the effect of a single and more intense noise exposure. In future studies, the present model could be used for studying age-related hearing loss in a more realistic situation. Possible intervention therapies may also be investigated in long-term studies using this model, aiming at better hearing preservation for the elderly.
